# Vowel production: a potential speech biomarker for early detection of dysarthria in Parkinson’s disease

**DOI:** 10.3389/fpsyg.2023.1129830

**Published:** 2023-08-28

**Authors:** Virginie Roland, Kathy Huet, Bernard Harmegnies, Myriam Piccaluga, Clémence Verhaegen, Véronique Delvaux

**Affiliations:** ^1^Metrology and Language Sciences Unit, Mons, Belgium; ^2^Research Institute for Language Science and Technology, University of Mons, Mons, Belgium; ^3^National Fund for Scientific Research, Brussels, Belgium

**Keywords:** Parkinson’s disease, dysarthria, early detection, acoustic analyses, vowel production

## Abstract

**Objectives:**

Our aim is to detect early, subclinical speech biomarkers of dysarthria in Parkinson’s disease (PD), i.e., systematic atypicalities in speech that remain subtle, are not easily detectible by the clinician, so that the patient is labeled “non-dysarthric.” Based on promising exploratory work, we examine here whether vowel articulation, as assessed by three acoustic metrics, can be used as early indicator of speech difficulties associated with Parkinson’s disease.

**Study design:**

This is a prospective case–control study.

**Methods:**

Sixty-three individuals with PD and 35 without PD (healthy controls-HC) participated in this study. Out of 63 PD patients, 43 had been diagnosed with dysarthria (DPD) and 20 had not (NDPD). Sustained vowels were recorded for each speaker and formant frequencies were measured. The analyses focus on three acoustic metrics: individual vowel triangle areas (tVSA), vowel articulation index (VAI) and the Phi index.

**Results:**

tVSA were found to be significantly smaller for DPD speakers than for HC. The VAI showed significant differences between these two groups, indicating greater centralization and lower vowel contrasts in the DPD speakers with dysarhtria. In addition, DPD and NDPD speakers had lower Phi values, indicating a lower organization of their vowel system compared to the HC. Results also showed that the VAI index was the most efficient to distinguish between DPD and NDPD whereas the Phi index was the best acoustic metric to discriminate NDPD and HC.

**Conclusion:**

This acoustic study identified potential subclinical vowel-related speech biomarkers of dysarthria in speakers with Parkinson’s disease who have not been diagnosed with dysarthria.

## 1. Introduction

Parkinson’s disease (PD) is a disease that causes degeneration of the nervous system, especially the substructures that control movement. This disease is characterized with the progressive loss of dopaminergic neurons. One of the strongest risk factors associated with disease is aging. Therefore, with the advancing age of the world’s population, the early detection of characteristic patterns of the disease is a health challenge. Moreover, as pointed out by Agüera-Ortiz et al. (2021), neurodegenerative diseases are an increased global economic and healthcare system burden.

The motor symptoms associated with PD may involve bradykinesia accompanied by resting tremor and/or rigidity. Patients with PD may experience symptoms that significantly affect their quality of life. Hypokinetic dysarthria, which includes a wide variety of speech disorders associated with PD, is one of them (Sapir, 2014; Postuma et al., 2015). Classical perceptual and acoustic studies have repeatedly shown that dysarthria affects the respiratory, phonatory and/or articulatory aspects of speech on both segmental and suprasegmental levels, i.e., dysprosody (e.g., Duffy, 2019). Dysarthric speech is then characterized by reduced loudness, monopitch and/or monoloudness, harsh voice, imprecise speech articulation or inappropriate silences. On the articulatory level, most of previous studies have focused on imprecision in consonant production (e.g., Ackermann and Ziegler, 1991) and vowel articulation (e.g., Skodda et al., 2011), in particular at moderate and advanced stages of the disease and for patients with moderate dysarthria (e.g., Martel-Sauvageau et al., 2015; Dias et al., 2016; Martel-Sauvageau and Tjaden, 2017; Duez et al., 2020). One of the most commonly reported impairments in individuals with PD who have hypokinetic dysarthria is difficulty in producing consonants accurately as typically evidenced by oral diadochokinetic tasks (e.g., Ackermann and Ziegler, 1991; McRae and Tjaden, 1998; Wong et al., 2011; Karlsson and Hartelius, 2019). The stops, affricates, and fricatives are often distorted, potentially due to the reduced range and strength of the movements used to produce them. Ackermann and Ziegler (1991), Ackermann et al. (1995) have suggested that this may be caused by PD patients trying to maintain a fluid speaking rate, at the risk of causing articulatory undershoot. However, research on muscle activity during speech in PD has yielded inconsistent results (for a review, see Walsh and Smith, 2012). For example, Mcauliffe et al. (2006) demonstrated that listeners perceived consonants as being produced with undershoot but did not find a corresponding reduction in tongue-palate contact on EPG examination. Wong Ackermann and Ziegler, 1991(2011) even found that some individuals with PD had increased distance of tongue movement when producing certain coronal and velar consonants. More research is needed to fully understand the dynamics of supra-laryngeal articulators in PD.

Interestingly, aspects of speech production related to sound resonance – central to the production of vowels, diphthongs and approximants – are thought to be preserved in PD, but have been little studied (Goberman et al., 2002). However, accurate execution of motor plans involving the jaw, tongue, and lips is as essential to the production of vowel-like sounds as it is to the production of consonants. The present study concerns vowel production in francophone speakers with PD. The study of vowel-like sounds, especially if it encompasses both stable and dynamically changing phases, could be a valuable area of research for better understanding speech motor control in PD. Indeed, adequate vowels and diphtongs can only be produced if one can both maintain stable articulatory configurations over time and properly execute dynamic sequences of coordinated articulatory gestures. Note that the available evidence about dysarthria in PD is often based on the English language although the variability across different languages should be considered in a speech assessment framework (Rusz et al., 2021). As it happens, English and French differ in their vowel inventory as well in the phonological structures associated with dynamic vocalic sounds: English counts a dozen vowels and several diphtongs, whereas the French inventory contains only monophtongs but also three approximants /w, ɥ, j/ resulting in sequences such as V.C_[glide]_V (kayak, brouillard, kiwi, etc.) (Fougeron and Smith, 1999).

A previous acoustic study was conducted on the speech productions of PD patients with mild dysarthria compared to healthy speakers (Delvaux et al., 2016). We specifically focused on the production of steady vowels and intervocalic glides, based on the hypothesis that parkinsonian speech production may be characterized by vowel centralization resulting in a reduction of the vowel space (Kent and Kim, 2003; Skodda et al., 2011; Mollaei et al., 2016). The study involved two groups of participants: 9 people (6 men and 3 women) with intermediate-stage Parkinson’s disease (according to the Hoehn and Yahr scale), and a healthy group of 10 people (5 men and 5 women) who had no speech or language disorders. Acoustic measurements were taken for sustained oral vowels, including overall duration and frequencies of formants (F1, F2) at the midpoint of the vowel, and individual triangular vowel space areas (tVSA) were calculated. Results showed that the mean areas did not differ significantly between the PD group and the control group. These results suggest that although there is more variation in the production of sustained vowels among persons with PD (here, with mild dysarthria), the size of their vowel spaces is not significantly different from those of HC. Other, complementary acoustic metrics would have to be used to capture subtle alterations in vowel production when dysarthria is mild.

In fact, a variety of acoustic metrics can help identify alterations in the productions of PD compared to HC speakers. In some studies, vowels metrics are calculated to identify a possible marker of the progression of the disease in PD (Sapir et al., 2010; Skodda et al., 2012; Rusz et al., 2013; Rountrey and Molett, 2020). The tVSA is one of the most frequently used acoustic indicator for the evaluation of imprecision in vowel production, as it can reflect major changes in articulatory movements in speech disorders. However, some researchers suggest that the tVSA is not sensitive enough to signal mild and moderate forms of dysarthria (Sapir et al., 2007; Neel, 2008; Skodda et al., 2011). Sapir et al. (2007) suggest that variations across speakers can statistically reduce the differences between those with mild dysarthria and those without dysarthria. Yet, a better understanding of the potential impairment in patients with mild dysarthria and those without dysarthria in PD is essential to identify speech deteriorations in the early stages of the disease. As far as we know, only the study conducted by Audibert and Fougeron (2012) proposes a direct comparison of several metrics derived from F1/F2 measurements to describe and quantify the possible distortions to be observed in the vowel space of French dysarthric speakers.

In the present study, we have selected three acoustic metrics which have been previously tested with PD patients for the complementary information they provide: (1) the triangular vowel space area (tVSA) representing the maximum working space of each individual, (2) the vowel articulation index (VAI), which is the reciprocal of the formant centralization ratio (Roy et al., 2009; Sapir et al., 2010) and (3) the PHI index which expresses the relationship between inter-category distance and intra-category variability within the vowel space considered as an organized system of phonemic categories (Huet and Harmegnies, 2000). Inter-category distance can be considered as a centralization metric and intra-category variability as an index of (in) consistency in the production of acoustic targets. Audibert and Fougeron (2012) suggest that metrics of intra-category dispersion and centralization are complementary. Their results show that intra-category variability is only weakly correlated with other metrics, arguing for its informational potential since it cannot be predicted by other measures.

Note that in clinical practice, the detection of alterations by an objective approach is intended to complete the perceptual analysis made by the clinician. In fact, the methodology of this study is designed to be applicable to a professional practice. The productions requested from the speakers are those that would be expected from a typical speech assessment by a speech therapist (recommendations by the American Speech-Language-Hearing Association, 2004). The number of productions per participant is intentionally limited, less than in typical experimental phonetics studies, which allows to eliminate a possible fatigue effect among participants.

Besides, while the majority of patients interviewed declare themselves dissatisfied with their communication performance (Miller et al., 2011), only a few individuals initiate speech therapy, even though available statistics tend to show an increase in speech therapy over the past few decades (Hartelius and Svensson, 1994; Kalf et al., 2011; Sunwoo et al., 2014; Schalling et al., 2017). Also, when it is present, speech therapy appears rather late in the course of dysarthria, with patients presenting moderate to severe dysarthria, whereas many recommendations suggest early speech therapy (Gentilhomme et al., 2020).

The purpose of this study is to use acoustic metrics to objectively identify speech biomarkers in oral vowel production in PD patients who do not have hypokinetic dysarthria, in order to identify speech alterations that are difficult to detect by even careful listening by the clinician. The long-term goal of this research is to identify early, subtle symptoms of dysarthria as a prodromal marker of PD. Indeed, recent evidence suggests that speech atypicalities might be the first motor signs to emerge (Sapir, 2014; Hlavnička et al., 2017; Rusz et al., 2021).

## 2. Methods

### 2.1. Participants

This study included 98 participants, divided into two groups. There were 63 participants diagnosed with idiopathic PD and 35 healthy controls (HC). The group of PD speakers was composed of Belgian French native speakers ranging in age from 38 to 85 years (mean age: 70), with an average disease duration of 7 years (ranging from 1 to 25 years) and representing all stages of Parkinson’s disease on the Hoehn and Yahr (1967) disability scale. All patients were diagnosed by the same neurologist following the UK Parkinson’s Disease Society brain bank criteria. Of the 63 participants with PD, 43 were dysarthric (DPD) and 20 were not dysarthric (NDPD) as determined by expert perceptual assessment during a complete speech assessment (respiratory aspects, articulatory aspects, oro-linguo-facial and pneumo-phono-articulatory coordination) and with the speech item (item 3.1) of the Movement Disorders Society-Unified Parkinson’s Disease Rating Scale part III/MDS-UPDRS (Goetz et al., 2008). All patients were evaluated by the same speech therapist during a speech assessment. The neurologist and the speech therapist are both specialized in the assessment and management of individuals with Parkinson’s disease. Both work in a day hospital department dedicated to individuals with PD.

Table 1 presents the characteristics of participants with PD in terms of sex, stage of disease (referring to Hoehn & Yahr stages), time since first diagnosis and dysarthria. It also provides scores on the original versions of UPDRS-III (motor score), Beck Depression Inventory/BDI-II (Beck et al., 1996), Montreal Cognitive Assessment/MoCA (Nasreddine et al., 2005), as well as the Parkinson’s Disease Questionnaire/PDQ-39 (Auquier et al., 2002) specific to quality of life.

**Table 1:** Characteristics of participants with PD in terms of sex, stage of disease, time since first diagnosis, dysarthria and scores of UPDRS-III, BDI-II, MoCA and PDQ-39.

Sexe	Stage (Hoehn and Yahr)	Duration_PD	UPDRS_III	Dysarthria	Severity_dysarthria	BECK (cut-off mild depression: 10–18)	MoCA (cut-off detecting MCI ≤ 25/30)	PDQ_39 (QoL deteriorated >50)
M	3	6	10	No	N/A	6	24	17
F	3	7	16	No	N/A	13	27	33
F	1,5	9	5	No	N/A	12	30	12
M	0	6	3	No	N/A	3	30	11
F	2,5	2	7	No	N/A	11	24	21
M	2	8	6	No	N/A	0	28	5
F	2,5	4	12	No	N/A	2	30	17
M	2,5	7	15	No	N/A	5	28	19
M	1	5	1	No	N/A	3	29	5
M	2	11	2	No	N/A	3	28	7
M	3	6	15	No	N/A	7	29	5
M	3	5	10	No	N/A	7	29	11
F	1,5	6	2	No	N/A	1	30	15
F	1,5	2	4	No	N/A	7	28	18
F	2	2	8	No	N/A	5	28	5
F	3	19	13	No	N/A	16	27	36
M	2	3	16	No	N/A	6	28	7
M	2	7	9	No	N/A	2	30	6
M	3	6	33	No	N/A	4	26	8
F	3	2	16	No	N/A	9	25	25
F	1,5	10	6	Yes	mild	6	29	26
M	2,5	13	15	Yes	moderate	6	27	13
M	3	24	4	Yes	mild	10	30	43
M	3	7	15	Yes	mild	11	29	19
M	4	2	45	Yes	moderate	8	30	23
F	4	7	20	Yes	mild	10	29	25
M	2	7	12	Yes	mild	11	27	14
F	2,5	11	11	Yes	mild	6	23	14
M	2,5	11	10	Yes	moderate	1	30	7
M	1,5	15	9	Yes	moderate	4	27	15
F	4	9	16	Yes	moderate	9	28	42
F	4	10	26	Yes	mild	12	27	37
F	1,5	9	7	Yes	moderate	8	30	21
M	2	3	16	Yes	moderate	25	26	30
F	2,5	3	13	Yes	mild	15	25	32
M	1,5	3	8	Yes	mild	5	29	12
M	2	3	1	Yes	moderate	0	29	3
M	3	6	35	Yes	mild	6	28	20
M	2,5	6	14	Yes	moderate	9	29	32
F	2,5	8	16	Yes	mild	15	30	30
F	3	9	20	Yes	moderate	9	28	23
M	1,5	2	11	Yes	mild	1	28	0
M	1,5	12	7	Yes	mild	1	30	9
M	1,5	6	7	Yes	mild	1	30	3
M	3	4	19	Yes	moderate	6	27	37
F	3	11	16	Yes	mild	11	26	30
F	2	8	5	Yes	mild	7	28	18
M	4	4	27	Yes	mild	18	24	49
M	3	4	18	Yes	mild	7	23	19
M	2	7	12	Yes	mild	3	26	7
F	4	15	35	Yes	mild	16	17	46
F	2	7	5	Yes	mild	2	28	3
F	5	18	48	Yes	moderate	10	21	46
M	4	6	14	Yes	mild	6	29	16
M	4	6	41	Yes	moderate	12	28	60
F	3	4	18	Yes	mild	6	30	21
M	1,5	1	5	Yes	mild	2	30	3
F	2,5	2	14	Yes	mild	11	27	25
F	2,5	2	13	Yes	mild	9	25	28
M	2	5	6	Yes	mild	11	26	24
F	1,5	5	8	Yes	moderate	8	27	13
M	1	4	2	Yes	mild	7	30	23
M	4	25	43	Yes	moderate	10	24	26

HC participants were aged 41 to 84 years (mean: 66) and presented nor reported any previous speech-language pathology.

### 2.2. Tasks

All PD patients were met in the ON (dopaminergic treatment) phase. Study participants were subjected to a variety of speech tasks, one of which was to repeat the cardinal French vowels/a, i, u/ five times. Steady oral vowels are the most easy-to-collect speech material in clinical settings. Furthermore, this production number allows for a compromise between clinical care and evaluation constraints while ensuring a sufficient number of repetitions to allow for robust statistical analysis of the collected data. Each participant thus performed fifteen isolated vowel productions. Only the results of this controlled task will be presented in this study. PD participants were assessed individually in a quiet room in the hospital and HC subjects were recorded under similar conditions, at home. The two groups were recorded with the same Zoom H5 portable recorder.

### 2.3. Acoustic measurements and acoustic metrics

Acoustic measurements were performed using Praat formant tracking and customized Praat scripts. The F1 and F2 values were obtained through a semi-automatic procedure from the steady state portion of each vowel. Specifically, the stable part of each vowel was manually identified based on information from the speech waveform and spectrogram, excluding unstable phases characterized by creaky voice, voicing interruption, breathing resumption, etc. The formant frequencies were automatically detected and manually verified, and their average value over the whole stable part was calculated.

Three different acoustic metrics were computed from the vowels produced by each speaker:

- The triangular Vowel Space Area (tVSA, in Hz^2^), which gives the size of the working vowel space for each participant (e.g., Kent and Vorperian, 2018). The tVSA is calculated using the formula:
$$\begin{array}{l} \mathrm{tVSA}=|0.5\times [\left(F2u+F2i\right)\times \left(F1u-F1i\right)-\left(F2a+F2u\right)\;\\ \;\;\;\;\;\;\;\;\;\;\;\;\;\;\;\times \left(F1u-F1a\right)-\left(F2a+F2i\right)\times \left(F1a-F1i\right)]| \end{array}$$

The higher the tVSA, the larger the participant’s vowel space.

- The Vowel Articulation Index (VAI), which concerns the tendency for vowel centralization, was developed by Sapir et al. (2010, 2011) to account for inter-speaker variability. According to these authors, since the measure of maximum vowel space is sensitive to inter-individual variability, the VAI allows to better represent any centralization of vowel formants. The goal of this index is to minimize sensitivity to interindividual variability and maximize sensitivity to vowel centralization with respect to tVSA (Sapir et al., 2010). Caverlé and Vogel (2020), in a study in which they compared several metrics to quantify vowel production (including tVSA and VAI), suggest that VAI is the most stable and sensitive measure under fatigue and noise conditions in healthy participants. According to Skodda et al. (2012), the VAI is considered to be a more effective measure than the Triangular Vowel Space Area (tVSA) for identifying speech difficulties in individuals with PD.

The VAI is calculated using the formula:

VAI=(F2i+F1a)∕(F1i+F1u+F2u+F2a)

The lower the calculated value, the higher the vowel centralization, and vice versa.

- The PHI index, which characterizes the level of organization of the vowel space, was calculated by determining the ratio between inter-category and intra-category dispersion within the vocalic system (Huet and Harmegnies, 2000). In addition to inter-category variability (e.g., variability due to vowel centralization), it can account for intra-category variability (e.g., variability due to vowel distortions). The phi index is the ratio between inter- and intra-categorical variability computed by analogy with the Fisher-Snedecor F-statistic in an analysis-of-variance model:
$$\Phi =\frac{\mathrm{inter}\_\mathrm{MS}}{\mathrm{intra}\_\mathrm{MS}}$$
Where: $$\mathrm{inter}\_\mathrm{MS}=\frac{\mathrm{inter}-\mathrm{category}\;\mathrm{sum}\;\mathrm{of squares}\;}{\mathrm{inter}-\mathrm{category degrees of freedom}\;}$$
And: $$\mathrm{intra}\_\mathrm{MS}=\frac{\mathrm{intra}-\mathrm{category}\;\mathrm{sum}\;\mathrm{of squares}\;}{\mathrm{intra}-\mathrm{category degrees of freedom}\;}$$

The inter-category mean square (inter_MS) is defined as the sum of the squares of the differences between the centroid of each vowel category and the general centroid of the entire vowel space, weighted by the number of vowels in each category and standardized by the total number of categories minus 1.

The intra-category mean square (intra_MS), on the other hand, is defined as the sum of the squares of the differences between each repetition of the same vowel and the centroid of the corresponding category, normalized by the number of vowels considered minus the number of categories.

Therefore, a lower PHI value suggests a lower degree of vocalic organization.

### 2.4. Statistical analysis

In order to assess the differences in acoustic parameters between PD patients and HC, statistical analyses were performed on all collected measurements using SPSS software (IBM SPSS Statistics 25). Because of the non-normality of the distributions non-parametric tests were chosen. Specifically, a series of Mann Whitney U tests were performed in order to make all possible pairwise comparisons between the three groups of participants.

## 3. Results

The demographic data (Table 1) allow us to observe a link between disease stage and motor symptoms (proportion of variance accounted η^2^: 0.677) and between disease stage and quality of life (η^2^: 0.468). Only a marginal fraction of the total variance was explained by the relationship between disease stages and time since first diagnosis (η^2^: 0.138). Moreover, no link is found between disease progression stages and presence/absence of dysarthria (η^2^: 0.047), nor between the presence/absence of dysarthria and disease duration (η^2^: 0.023), depressive symptoms (η^2^: 0.040), cognitive impairment (η^2^: 0.012), or quality of life (η^2^: 0.098).

The proportion of participants did not differ significantly in the groups either in terms of sex (Pearson chi-square test, *χ*^2^ = 0.075; *p* = 0.785) or age (*χ*^2^ = 43.151; *p* = 0.298).

### 3.1. Triangular vowel space area

The calculation of the triangular vowel space area (tVSA) showed that on average, the mean area was significantly smaller for DPD patients than for HC participants (U = 1,400, *p* = 0.027). The area values were significantly greater for HC participants (mean: 363679 Hz^2^) compared to those in the DPD group (mean: 306501 Hz^2^), except for the first repetition. Indeed, when the five iterations per vowel produced by the participants were considered separately, we found that, the first production of the phonemes /a, i, u/ had similar characteristics in both groups. The four other productions were significantly different between the two groups.

However, these differences were only found for DPD speakers compared to HC speakers. No differences were observed between the productions of NDPD and HC participants. We also observe no significant differences between the productions of DPD and NDPD participants, which is in contradiction with the distinction made by clinicians regarding the presence or absence of dysarthric symptoms in these patients. Overall, we also observe a high interindividual variability in PD speakers.

### 3.2. Vowel articulation index

The VAI values were found to be significantly different between DPD patients and HC speakers (U = 1,519, *p* = 0.001), indicating that the dysarthric speakers with PD had more centralized vowel productions and less contrast between vowels when compared to HC participants.

As observed from the tVSA metric, we were unable to identify differences between NDPD and HC participants from the VAI centralization index. However, unlike the results obtained from the calculation of the tVSA metric, the VAI centralization index allows us to uncover significant differences between the productions of the DPD and NDPD speakers.

### 3.3. Index of the level of organization of the vowel space (PHI)

Regarding the PHI index, there was no difference between the productions of DPD and NDPD speakers. However, the PHI values were found to be significantly higher for HC speakers (mean: 1477) compared to DPD patients (mean: 150) (U = 1960, *p* < 0.001). Indeed, a high level of formant centralization was observed in DPD speakers, resulting in lower inter-category differentiation than in HC speakers (U = 1,511, *p* = 0.001). Furthermore, intra-category dispersion was significantly lower in HC speakers than in the DPD group (intra_MS: mean: 8751 vs. mean: 31125; U = 278, *p* < 0.001).

**Figure 1:**
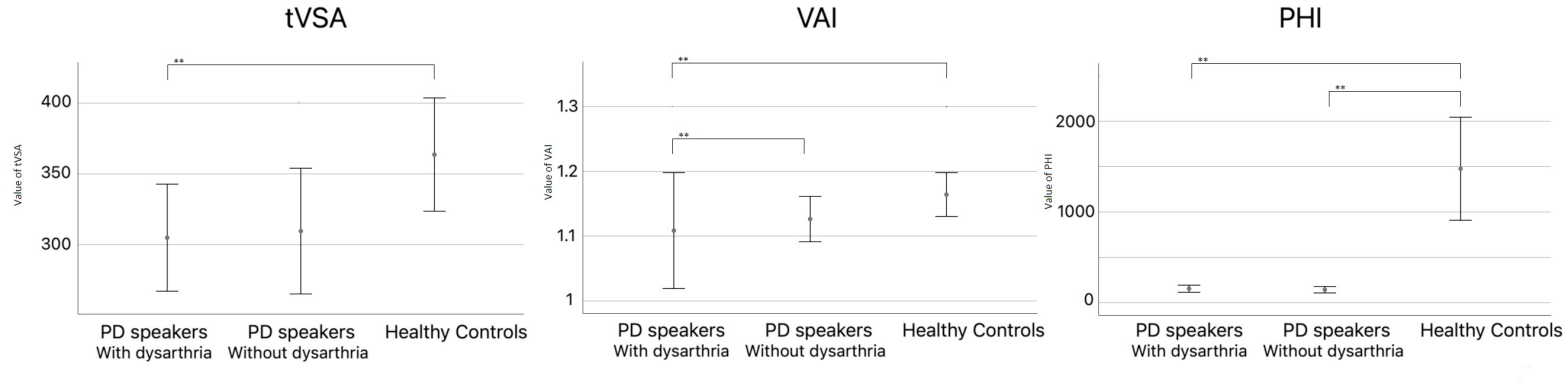
Mean tVSA [KHz^2^], VAI and PHI across the three groups of participants. Error bars represent 95% confidence intervals. Significant pairwise comparisons are represented by asterisks (** 0.01 significant threshold).

The PHI metric also showed a significant difference between NDPD and HC speakers (U = 639, *p* < 0.001). This difference was primarily due to a higher intra-categorical dispersion in NDPD patients, likely resulting from larger variability in vowel production (U = 86, *p* < 0.001) (see Figure 1).

## 4. Discussion

The purpose of this study was to identify objective vocal biomarkers in the production of oral vowels among parkinsonian speakers. The aim was to support the clinicians in identifying subtle acoustic alterations that may be difficult to detect perceptually, in order to allow an early diagnosis of dysarthria, even when clinical symptoms are subclinical. Furthermore, the relationships between PD and dysarthria are not bidirectional: not all Parkinson’s patients necessarily develop dysarthria, and the presence and severity of dysarthria can vary from one patient to another and evolve at a different pace than the progression of the disease (Dias et al., 2016; Karan et al., 2022). Moreover, the analysis of demographic data highlights a lack of correlation between the progression of the disease (disease stages and duration since diagnosis) and the presence or absence of dysarthric symptoms. Therefore, the sole progression of the disease does not appear to be a reliable indicator of the progression of dysarthria.

Through an acoustic analysis of the productions of the vowels /a, i, u/, we computed three acoustic metrics considered as complementary because of the information they provide: information on the maximum vowel working space (tVSA), information on the accuracy during the productions (PHI, and more particularly intra_MS, the intra-category variability), information on a possible phenomenon of centralization of the vowel targets (VAI as well as inter_MS, the component of the PHI metric that reflects inter-category variability).

Using these combined metrics, the overall goal was to better identify global variations in the exploitation of the vowel system in the three groups of participants. The results demonstrate the benefits of combining several acoustic metrics to characterize the vowel system of PD speakers. First, the tVSA metric, which is the most frequently used in research on the vowel system in pathological speech, enables to uncover alterations in DPD speakers compared to HC speakers. In fact, both groups of speakers had similar tVSA values for the first repetition of the phonemes /a, i, u/, but differed significantly for the other four productions, DPD speakers exhibiting smaller vowel space areas than healthy controls. This result pattern can be interpreted as DPD speakers transiently resorting to hyperarticulation (relative to their own routines) on their first attempt to repeat the vowel. The significant differences observed on subsequent repetitions suggest that they could not maintain this strategy for the remainder of the vowel sequence.

Importantly, tVSA does not allow to distinguish NDPD speakers from parkinsonian participants with dysarthria or from healthy speakers. Thus, this metric is not sensitive enough to identify subclinical manifestations of dysarthria, supporting Skodda et al. (2011) suggestion of a low informative potential of tVSA in detecting slight changes during vowel production by PD speakers. The lack of significant differences in our study between DPD and NDPD in terms of tVSA would result from the fact that dysarthria-related alterations in steady-state vowel production are too subtle to be highlighted by tVSA calculation.

Second, the VAI centralization metric is valuable in that it reflects the categorization made by the speech therapists during speech assessment between PD patients with and without dysarthria. These findings which corroborates the perceptual distinction between the groups as formulated by the speech therapist, in accordance with Skodda et al. (2011), suggest that speech therapists may use vowel centralization as a cue of dysarthria, i.e., a form of hypoarticulation characterized by a general shift of vowel targets toward the center of the vowel space. However, this metric does not appear to be useful in searching for potential early, subtle speech alterations that might distinguish NDPD speakers from HC, which suggests that NDPD speakers produce vowels as dispersed in the vowel space as those of typical, healthy participants.

Third, the PHI metric yields very different results depending on whether participants are healthy controls or Parkinsonian participants (both with and without dysarthria). PHI values were found to be significantly lower for parkinsonian speakers which indicates that their vocalic system is substantially less organized than that of control speakers.

For DPD speakers, inter-category dispersion was reduced and intra-category variability was increased. Significantly lower inter-category dispersion is in line with higher centralization, in accordance with the results of the VAI metric for these speakers. Greater intra-category variability suggests difficulty in repeatedly producing the same vowel in the same way, which may reflect articulatory instability and/or more variable speech targets.

As to NDPD speakers, PHI was the only metric that showed a significant difference between their vocalic productions and those of healthy speakers. However, this difference was primarily due to a higher intra-categorical dispersion in NDPD patients, likely resulting from larger variability in vowel production. Therefore, what was significantly reduced among NDPD speakers was not so much the overall articulatory range/workspace (indexed by tVSA), but the internal organization of the vowel system itself due to the lack of accuracy around vowel targets.

Unlike the other two metrics, PHI accounts for intra-category variability (intra_MS) in vowel production, which appears to be substantially increased for all PD participants, even for those who have not been diagnosed with dysarthria.

In summary, following our acoustic analyses based on a diversity of metrics, we confirm in the present study the presence of potential speech biomarkers of dysarthria in NDPD. The PHI metric could be considered a potential biomarker for the early stages of dysarthria in people with PD as it is the only measure capable of detecting subtle differences in vowel production between NDPD and HC speakers,

even though it does not allow for the differentiation of DPD and NDPD speakers. Those differences reside in larger intra-categorical variability presumably due to a difficulty in reaching vowel targets with accuracy and consistency. Such alterations seem to occur in the initial stages of PD, or at least when the dysarthria is still subclinical, which is in line with recommendations for early evaluation of dysarthria in PD, so that early speech therapy can be considered.

It should be noted that the limited number of data points collected per speaker should be considered, in our opinion, not as a limitation, but as an asset of the present study. Indeed, our goal was to propose an analysis based on a procedure that could be easily integrated into the clinical practice of speech therapists. Faced with a clinical problem, the intention is to propose an early detection method for a systematic screening of hypokinetic dysarthria with a semi- automatic acoustic analyses routine. Such semi-automatic screening procedures involving manually supervised acoustic measures to be integrated into clinical practice of speech therapists are currently tested in the framework of the MonPaGe protocol so that they require no more than a few minutes of analysis per patient for the clinician, the intervention of the speech therapists required to check the automatic segmentation as well as adjusting some key parameters (Laganaro et al., 2021).

Among the limitations of the present study, the most significant one concerns the evolution of NDPD patients. A longitudinal study confirming or refuting the subsequent appearance of dysarthric symptoms would allow us to reinforce or qualify our results.

Moreover, we ensured that the relative proportions of men and women in each group were identical to ensure the relevance of comparisons between groups even though the data was not standardized. Examining the effects of normalizing formant values, as recently proposed by Kuo and Berry (2023), may be relevant to a future study.

Furthermore, the characteristics of our PD patients allow us to identify participants with mild cognitive impairment (*N* = 11). However, the results on the MoCA do not appear to be correlated with the presence/absence of dysarthria (η^2^ = 0.012). A future study focusing on the effects of cognitive impairments and the progression of dysarthria in Parkinson’s disease could be conducted, as speech motor control requires significant cognitive resources.

The perspectives of the present work relate to the potential value of the PHI index for the differential diagnosis of Parkinson’s disease. Currently, we are conducting an acoustic analysis of spontaneous vowels produced by the same participants in a picture description task. The objective is to consolidate the findings of the present study concerning the interest of the PHI index for the detection of subtle, subclinical speech alterations in PD, i.e., even in patients without dysarthria. Next, we will recruit patients in the diagnostic phase as well as previously diagnosed patients in order to identify biomarkers that can be used to guide the diagnosis of PD vs. other related pathologies (e.g., Parkinson +, progressive supranuclear palsy, multiple system atrophy). Indeed, there are still few studies comparing the productions of these patients for differential diagnosis purposes as highlighted by Daoudi et al. (2022).

## Data availability statement

The raw data supporting the conclusions of this article will be made available by the authors, without undue reservation.

## Ethics statement

The studies involving human participants were reviewed and approved by CHU-Charleroi and Erasme-ULB (P2015/527/B406201526528). The patients/participants provided their written informed consent to participate in this study.

## Author contributions

All authors listed have made a substantial, direct, and intellectual contribution to the work and approved it for publication.

## Funding

This study has partially benefited from the funding attributed to the Research Project « Parolpathos », Action de Recherche Concertée AUWB- 2012-12/17-UMONS-N°1.

## Conflict of interest

The authors declare that the research was conducted in the absence of any commercial or financial relationships that could be construed as a potential conflict of interest.

## Publisher’s note

All claims expressed in this article are solely those of the authors and do not necessarily represent those of their affiliated organizations, or those of the publisher, the editors and the reviewers. Any product that may be evaluated in this article, or claim that may be made by its manufacturer, is not guaranteed or endorsed by the publisher.
